# Structure of the Toll/Interleukin-1 Receptor (TIR) Domain of the B-cell Adaptor That Links Phosphoinositide Metabolism with the Negative Regulation of the Toll-like Receptor (TLR) Signalosome*[Fn FN2]

**DOI:** 10.1074/jbc.M116.761528

**Published:** 2016-12-01

**Authors:** Samer Halabi, Eiki Sekine, Brett Verstak, Nicholas J. Gay, Martin C. Moncrieffe

**Affiliations:** From the Department of Biochemistry, Cambridge University, Cambridge CB2 1GA, United Kingdom

**Keywords:** crystal structure, MAL/TIRAP, phosphatidylinositide 3-kinase (PI 3-kinase), phospholipase C, Toll-like receptor (TLR), BCAP, pathway crosstalk, BCAP adaptor, TIR domain, PI3 kinase, PLCγ2, phosphatidylinositol 4,5-bisphosphate, Toll-like receptor

## Abstract

Ligand binding to Toll-like receptors (TLRs) results in dimerization of their cytosolic Toll/interleukin-1 receptor (TIR) domains and recruitment of post-receptor signal transducers into a complex signalosome. TLR activation leads to the production of transcription factors and pro-inflammatory molecules and the activation of phosphoinositide 3-kinases (PI3K) in a process that requires the multimodular B-cell adaptor for phosphoinositide 3-kinase (BCAP). BCAP has a sequence previously proposed as a “cryptic” TIR domain. Here, we present the structure of the N-terminal region of human BCAP and show that it possesses a canonical TIR fold. Dimeric BCAP associates with the TIR domains of TLR2/4 and MAL/TIRAP, suggesting that it is recruited to the TLR signalosome by multitypic TIR-TIR interactions. BCAP also interacts with the p85 subunit of PI3K and phospholipase Cγ, enzymes that deplete plasma membrane phosphatidylinositol 4,5-bisphosphate (PIP2), and these interactions provide a molecular explanation for BCAP-mediated down-regulation of inflammatory signaling.

## Introduction

Phosphoinositide 3-kinases (PI3Ks) are crucial mediators of various cellular processes including growth, survival, proliferation, migration, metabolism, and membrane transport. Dysfunction in the PI3K signaling network is a major driver of oncogenesis (1–4). The well characterized class Ia PI3Ks phosphorylate primarily at the D-3 position of the inositol ring, creating a site that enables the interaction of proteins with the plasma membrane. Class Ia PI3Ks are heterodimers comprised of regulatory (p85, p55, or p50) and catalytic (p110) subunits (5). The regulatory subunit not only represses the activity of the catalytic subunit in unactivated cells but mediates its activation by interacting directly with phosphorylated tyrosine residues present on activated growth factor receptors or adaptor molecules.

One such adaptor, B-cell adaptor for phosphoinositide 3-kinase (BCAP),^4^ functions in linking the B-cell receptor (BCR) and the co-receptor CD19 to the activation of PI3K via interaction with the SH2 domains on the regulatory p85 subunit (6). Architecturally, BCAP contains well defined ankyrin repeats and a Dof/BANK/BCAP (DBB) domain; the latter was first reported in the *Drosophila* protein Dof, where it is required for FGF-dependent signaling (7, 8), and a homologous domain exists in the B-cell scaffold with ankyrin repeat (BANK) protein (9).

The expression and cellular distribution of BCAP suggest that it is involved in immunological processes in addition to regulation of BCR (10, 11). In mice, BCAP is specifically expressed not only in B-cells but also in macrophages, dendritic cells, and lymphocyte natural killer cells (13). BCAP is mainly localized in the cytosol and the plasma membrane of expressing cells, and there are two splice variants in chicken, humans, and mice (6). Full-length BCAP is designated BCAP_L_, and the second largest splice variant is designated BCAP_S_.

BCAP_L_ is recruited into activated complexes of Toll-like receptors (TLRs) and associated signaling adaptor proteins. TLRs are pattern recognition receptors that respond to microbial products such as lipopolysaccharide from Gram-negative bacteria. The TLRs and the signaling adaptors MyD88 and MAL/TIRAP contain TIR domains, and these interact via homo- or heterotypic interactions to form complexes that constitute the post-receptor signalosome. A recent study predicted the existence of a TIR domain in the N-terminal region of BCAP_L_ that is required for negative regulation of inflammatory signaling by TLRs (14). RNAi depletion of BCAP_L_ from macrophages enhanced the LPS-induced production of NF-κB and the inflammatory cytokines IL-6 and IL-10 but not TNF-α (10), which suggests that it negatively regulates inflammation. Similar results were obtained upon stimulation of TLR2/6, TLR3, and TLR7 by their respective ligands (10). In contrast, RNAi-mediated depletion of BCAP_S_, a splice variant that lacks the putative TIR domain, is a positive regulator of inflammation (10).

Crosstalk between the TLR and PI3K pathways could be facilitated by the TIR domains on the TLR receptors or any of the well characterized TLR adaptor proteins TIRAP, MyD88, TRIF, TRAM, or SARM. However, the situation is more complex. Tyrosine phosphorylation of the Y*XX*M motif on BCAP, the first of which lies outside of the putative TIR domain, enables interaction with the SH2 domain of p85 (6, 12, 15). Additionally, isolated B-cells from BCAP knockdown mice exhibited decreased calcium mobilization, which was attributed to a decrease in the activation of the enzyme PLCγ2 (16). PLCγ2, like p85, has both SH2 and SH3 domains, and its decreased activation may be the result of direct interaction with BCAP (16). The PLCγ2 signaling pathway has been implicated in TLR4 signaling (17), but the molecular mechanisms and interactions are poorly understood.

Here we present the structure of the N-terminal domain of BCAP, firmly establishing it as a TIR domain. We also show that full-length BCAP is dimeric and that its oligomerization is dependent on its ANK and DBB domains. Furthermore, we show that BCAP TIR associates with the MAL/TIRAP adaptor and the TIR domains of TLRs. BCAP also binds to PLCγ2, and this interaction does not inhibit its association with the tyrosine kinases Syk and Lyn. The interaction between BCAP and the TLRs, as well as its interaction with PI3K and PLCγ2, suggests that BCAP plays a central role in crosstalk between the TLR, PI3K, and PLCγ2 pathways.

## Results

### 

#### 

##### Structure of the N-terminal TIR Domain of Human BCAP

**FIGURE 1.**
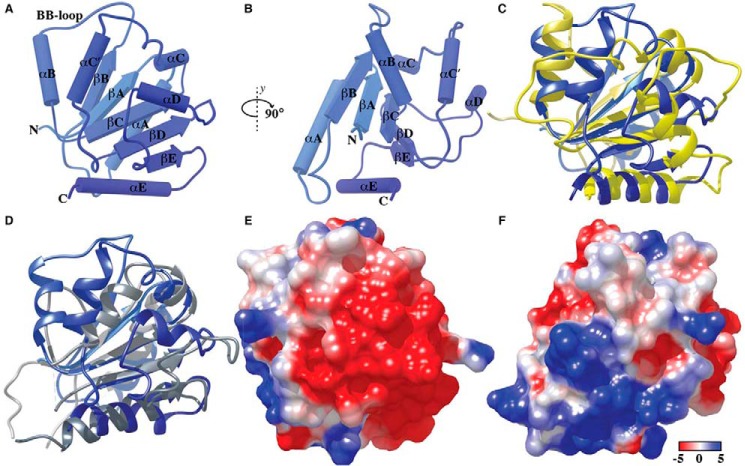
**Structure of the TIR domain of human BCAP.** *A* and *B*, the BCAP TIR domain has the typical TIR fold consisting of five parallel β-strands βA–βE) surrounded by five helices (αA–αE). *C* and *D*, structural superposition of BCAP-TIR and the TIR domain of TLR2 (*C*) and MAL/TIRAP (*D*). Electrostatic surface potential of BCAP-TIR (*E* and *F*) using the orientations shown in *panels A* and *B* is colored from −5 (*red*) to +5 kcal/mol (*blue*). Electrostatic potentials were calculated using Chimera.

The N-terminal domain of BCAP comprising residues 7–142 was solubly expressed in *Escherichia coli*. The structure was solved at 2.5 Å using data derived from protein crystals in which the lysine residues were dimethylated and single-wavelength anomalous dispersion phasing with 5-amino-2,4,6-triiodoisophthalic acid monohydrate (18). Data collection, phasing, and refinement statistics are summarized in supplemental Table 1. There was a single molecule in the asymmetric unit, and the structure reveals that the N-terminal region adopts a typical TIR domain fold (Fig. 1*A*). The TIR domain of BCAP is composed of five parallel β-strands (βA–βE) surrounded by five α-helices(αA–αE). The third α-helix has a kink and forms two helices, αC and αC′, and the fourth is distorted. The loop regions, with the exception of the biologically important BB-loop, which connects the βB-strand to the αB-helix, were well defined in the electron density, and unlike the structure of the MAL-TIR domain (19), there are no disulfide bonds.

Using the PDBeFOLD (20) and VAST (21) servers, the closest structural homologues were both TLR2-TIR domain mutants (22, 23) (Protein Data Bank (PDB) codes 1FYX and 1O77, respectively), although the sequence identity between the TLR2-TIR domain and BCAP-TIR is only 12%. Other close homologues include the TIR domain of the interleukin-1 receptor, IL-1RAPL (24), as well as MAL/TIRAP (19, 25) and the TIR domain of TLR10 (26). Secondary structural alignment of the BCAP TIR domain to its closest structural homologues is shown in Fig. 1, *C* and *D*. The BB-loop in BCAP is longer and not as rigid as that in TLR2. BCAP is less compact than MAL/TIRAP, which lacks the BB-loop and also contains two disulfide bonds. Strikingly, the orientation of the βB-sheet and the αB-helix of BCAP TIR domain is such that it extends out of the domain core in comparison with that of MAL/TIRAP. With regard to sequence conservation across the structurally homologous TIR domains, there are five highly conserved residues including Ser-17 (αA-loop), Leu-32 (αA), Pro-49 (βB), Leu-101, and Leu-102 (βD) (supplemental Fig. 1). The electrostatic potential of the solvent-accessible surface of BCAP (Fig. 1, *E* and *F*) reveals distinct regions of negative and positive potential in addition to hydrophobic patches, suggesting that the TIR domain of BCAP may interact with diverse TIR domain-containing proteins. For example, the TIR domain of MAL/TIRAP is predominantly hydrophobic but has acidic and small basic patches (19), which could, in principle, interact with acidic or basic surfaces on BCAP-TIR. In contrast, the TIR domain of MyD88 has significant regions of basic residues that may interact with the acidic surface on the TIR domain of BCAP.

Given the necessity of TIR-TIR interactions to signal propagation, several mutations were made to explore possible regions on BCAP that may be involved in its association with MAL/TIRAP. Sequence alignments of BCAP and proteins known to interact with MAL/TIRAP (MyD88, p85, A46, and TRAF6) revealed three conserved residues (Leu-70, Asp-117, and Leu-401). Two of these (Leu-70 and Asp-117) lie within the TIR domain, and all were mutated to alanine. Additionally, the highly conserved βB loop proline residue (Pro-49), which directs adaptor specificity in TLRs (27), was mutated to aspartic acid and alanine, respectively. The co-immunoprecipitation data for these mutants and MAL/TIRAP (supplemental Fig. 3) suggest that Pro-49 is not required for the interaction. Both the D75A and L401A mutations exhibited reduced binding, whereas the D117A mutation abolishes the interaction between BCAP and MAL/TIRAP. Asp-117 is located in a region of acidic surface potential sandwiched between the αD-helix and βE-sheet, and it is likely that this region interacts with a basic patch on MAL/TIRAP. The reduced association observed with the L401A mutant suggests that the determinants of the BCAP-MAL/TIRAP interaction may not solely reside within the TIR domain, and it is likely that there are other regions involved in the BCAP-MAL/TIRAP hetero-association.

##### Full-length BCAP Is Dimeric, but the TIR Domain Is Monomeric

**FIGURE 2.**
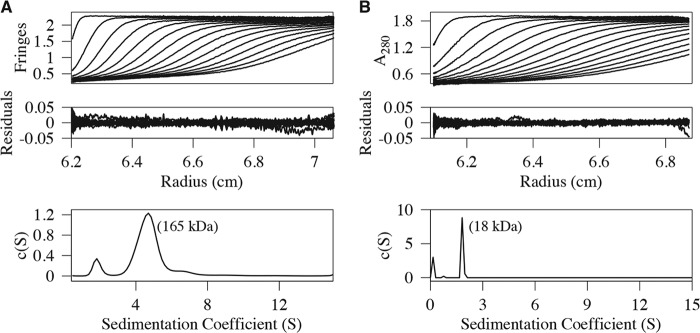
**AUC analysis of full-length BCAP and TIR domain.** *A* and *B*, sedimentation velocity data, residuals, and the sedimentation coefficient distribution, c(S), for full-length BCAP (*A*) and the N-terminal region comprising residues 7–142 (*B*). The recovered molecular masses from a c(M) analysis are also shown. The frictional coefficient was well determined, and as a result, the molecular masses are reliable. Full-length BCAP is dimeric, and the N-terminal domain is monomeric. Every third scan is shown for clarity.

Analytical ultracentrifugation (AUC) experiments were performed on purified samples of full-length BCAP_L_ and an N-terminal region spanning residues 7–142, which contains the TIR domain. The sedimentation velocity data, the residuals after fitting, and the c(S) distribution are shown in Fig. 2. The sedimentation coefficient values were 4.70 × 10^−13^ *s* and 1.82 × 10^−13^ *s* for BCAP_L_ and the N-terminal domain, respectively. The qualities of the fits were very good with root mean square deviation values of 0.007 (BCAP_L_) and 0.005 (N-terminal domain). Additionally, the frictional coefficient was well determined, and this allows estimation of molecular masses from the sedimentation velocity data. Using c(M) analyses, the molecular masses are 165 and 18 kDa for BCAP_L_ and the TIR domain, respectively. These values are within ∼10% of those expected for dimeric BCAP_L_ and monomeric N-terminal domain on the basis of their amino acid sequence. Weight-averaged molecular masses obtained from an analysis of the SEC-MALS (supplemental Fig. 2, *A* and *C*) data are consistent with those obtained by AUC.

##### The DBB and ANK Domains Mediate BCAP Oligomerization

**FIGURE 3.**
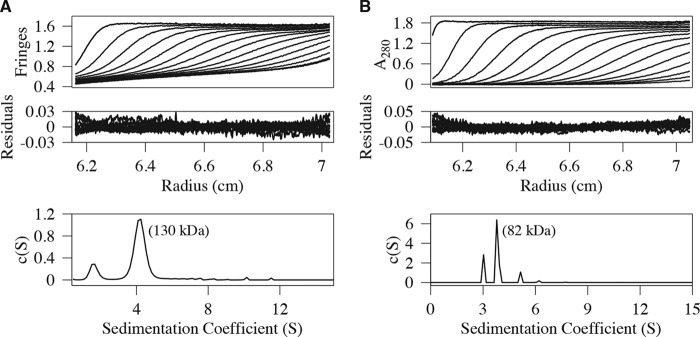
**AUC analysis of BCAP splice variant and N-terminal variant.** *A* and *B*, sedimentation velocity profiles, residuals, and sedimentation coefficient distribution, c(S), for the splice variant BCAP_S_, which lacks the first 180 amino acids and contains the DBB domain and ANK repeats (residues 322–403) (*A*), and BCAP_330_, which consists of the N-terminal 330 residues and includes the putative TIR and DBB domains (*B*). Both are dimeric, revealing that the DBB and ANK domains are responsible for BCAP_L_ self-association. Every third scan is shown for clarity.

The monomeric nature of the TIR domain implies that the determinants of BCAP_L_ self-association lie outside this region. To clearly define the regions responsible for BCAP_L_ oligomerization, the splice variant (BCAP_S_) and a truncated molecule comprising the first 330 residues of BCAP_L_ (BCAP_330_), which contains the TIR and DBB domains, were expressed, and their hydrodynamic behavior was studied by sedimentation velocity. Fig. 3 shows an analysis of the sedimentation velocity data for these constructs, and both, like BCAP_L_, are dimeric. SEC-MALS data for BCAP_330_ (supplemental Fig. 2*B*) also confirm that it is dimeric. Because BCAP_330_ contains the DBB domain, this confirms that it is, at least in part, responsible for the self-association of BCAP_L_. The ankyrin repeats are also known to facilitate oligomerization (28), and together with the DBB domain, they may enhance self-association.

##### Human BCAP Interacts with MyD88, MAL/TIRAP, TLR2, TLR4, and SARM but Not TRAM

**FIGURE 4.**
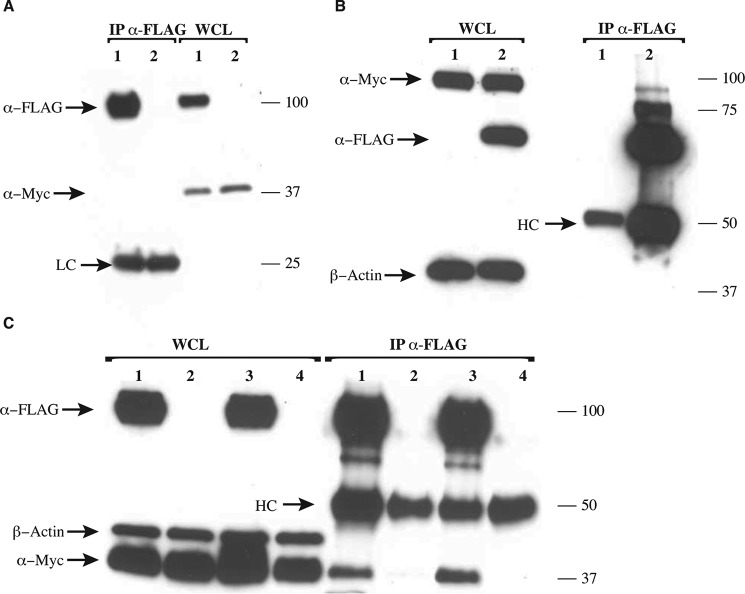
**Interaction of BCAP and other TIR domain-containing proteins.** HEK 293T cells were overexpressed with Myc-TRAM, FLAG-BCAP, Myc-BCAP, FLAG-SARM, Myc-MyD88, and a Myc-MyD88-F*XX*M mutant as indicated. Cells were lysed, and immunoprecipitation (*IP*) was performed using anti-FLAG antibody. Precipitates were split and assayed for the presence of FLAG- or Myc-tagged proteins. *A*, BCAP shows no interaction with TRAM (*lane 1*, Myc-TRAM+FLAG-BCAP-FL; *lane 2*, Myc-TRAM). *WCL*, whole cell lysate. *B* and *C*, BCAP interacts with SARM (*lane 1*, Myc-BCAP-FL; *lane 2*, Myc-BCAP-FL and FLAG-SARM) (*B*) and with both MyD88 and the F*XX*M mutant of MyD88 (*lane 1*, Myc-MyD88-WT and FLAG-BCAP-FL; *lane 2*, Myc-MyD88-WT; *lane 3*, Myc-MyD88-F*XX*M and Myc-BCAP-FL; *lane 2*, Myc-MyD88-F*XX*M) (*C*). *HC* and *LC* are the heavy and light chains of the mouse anti-FLAG antibody, respectively.

TIR domains associate via homotypic interactions, and the presence of an N-terminal TIR domain in BCAP could potentially enable binding with the cytosolic adaptor proteins involved in the TLR pathway. To determine whether BCAP_L_ interacts with TRAM, SARM, and MyD88, pulldown assays were performed. HEK 293T cells were transiently transfected with Myc-TRAM, FLAG-SARM, Myc-MyD88, and Myc-MyD88-F*XX*M mutant in the presence of either Myc-BCAP or FLAG-BCAP. BCAP showed no interaction with Myc-TRAM (Fig. 4*A*) but interacted with FLAG-SARM (Fig. 4*B*) and also with Myc-MyD88 and the Myc-MyD88-F*XX*M mutant (Fig. 4*C*).

The nature of the interaction between BCAP and TIRAP was also probed by a yeast two-hybrid assay utilizing a construct consisting of the first 434 amino acids of BCAP (BCAP-TDA). This construct includes the dimerization site, two of the four Y*XX*M motifs present in the full-length protein, and the TIR, DBB, and ANK domains. BCAP-TDA was tested against full-length constructs of MAL/TIRAP and its mutants D96N and S180L, the TIR domains of TLR2 and TLR4, and also the regulatory p85 subunit of PI3K. In these experiments, all proteins tested interacted with BCAP-TDA (see supplemental Fig. 4). Interestingly, the disease-associated MAL/TIRAP variants S180L (29) and D96N (30) also bind BCAP-TDA, strongly suggesting a different binding mode to that proposed for MyD88 (19).

##### BCAP Associates with PLCγ2, Lyn, and Syk

BCAP_L_ is involved in the BCR-mediated activation of PLCγ2 and PI3K (6). The interaction between BCAP_L_ and PI3K is direct and is known to involve at least one of two SH2 domains on the regulatory p85 subunit of PI3K. The p85 subunit also has an SH3 domain that is separated from the first N-terminal SH2 domain by a Rho-GAP domain (5, 31), and it is not known whether this domain is involved in the interaction with BCAP. Like p85, PLCγ2 contains two SH2 domains and an SH3 domain, and given the similarities in domain structure, we hypothesized that BCAP could interact with PLCγ2 in a manner analogous to its interaction with the p85 subunit. Additionally, there is ambiguity regarding the identity of the kinase that activates PLCγ2, with both Syk and Btk having been implicated (17, 32).

**FIGURE 5.**
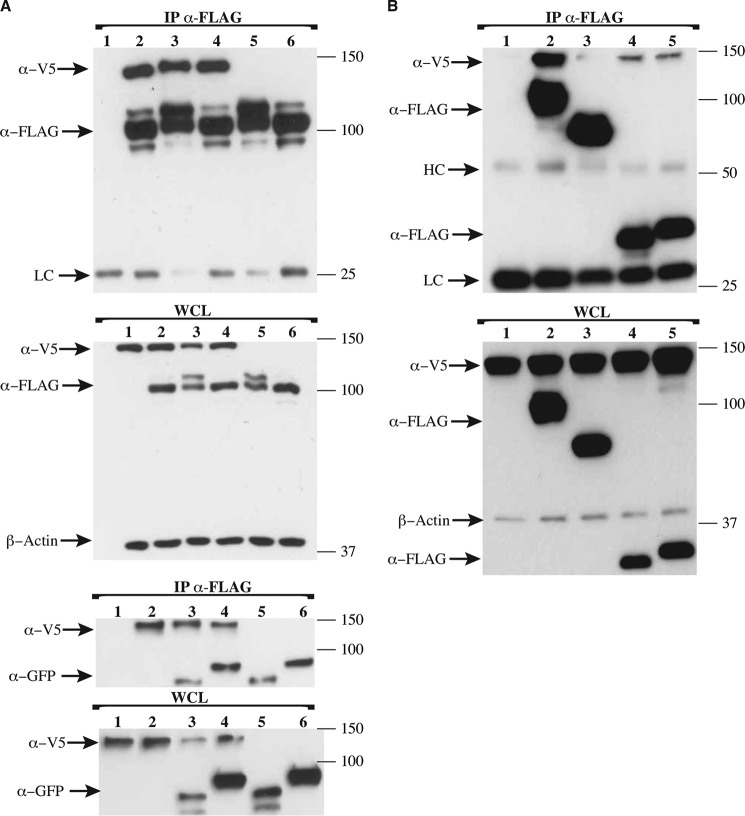
**BCAP associates with phospholipase C**γ**2 and the kinases Lyn and Syk.** HEK 293T cells were overexpressed with PLCγ2-V5, FLAG-BCAP, Lyn-GFP, YFP-Syk, and TIRAP-FLAG. Cells were lysed, and immunoprecipitation was performed using anti-FLAG antibody. Precipitates were split and assayed for the presence of V5-, FLAG-, GFP-, and YFP-tagged proteins as shown. *A*, the interaction between BCAP and PLCγ2 is independent of the tyrosine kinases Syk and Lyn; BCAP associates with Syk or Lyn both in the presence and in the absence of PLCγ2 (*lane 1*, PLCγ2-V5; *lane 2*, PLCγ2-V5 and FLAG-BCAP-FL; *lane 3*, PLCγ2-V5, FLAG-BCAP-FL, and GFP-Lyn; *lane 4*, PLCγ2-V5, FLAG-BCAP-FL, and YFP-Syk; *lane 5*, FLAG-BCAP-FL and GFP-Lyn; *lane 6*, FLAG-BCAP-FL and YFP-Syk). *B*, both MAL/TIRAP and MyD88 show reduced association with PLCγ2 relative to BCAP, whereas SARM does not interact with PLCγ2 (*lane 1*, PLCγ2-V5; *lane 2*, PLCγ2-V5 and FLAG-BCAP-FL; *lane 3*, PLCγ2-V5 and FLAG-SARM; *lane 4*, PLCγ2 -V5 and TIRAP-FLAG; *lane 5*, PLCγ2 -V5 and MyD88-FLAG). The expression of β-actin was used to assess the relative expression levels of the transformed plasmids. *HC* and *LC* are the heavy and light chains of the mouse anti-FLAG antibody, respectively.

To address these questions, pulldown assays using BCAP_L_ and human PLCγ2 (PLCγ2-V5) were carried out in the presence or absence of either human YFP-tagged Syk or GFP-tagged Lyn. The results presented in Fig. 5*A* show that FLAG-BCAP_L_ associates with PLCγ2-V5 in the presence and absence of either YFP-Syk or GFP-Lyn. Moreover, the addition of Syk or Lyn did not increase the apparent binding of BCAP_L_ to PLCγ2, suggesting that the interaction between BCAP and PLCγ2 may be promoted by endogenous tyrosine kinases in these experiments. However, it is apparent from the presence of a higher molecular mass band of BCAP that it is phosphorylated by the transfected Lyn kinase. BCAP was also able to pull down both Lyn and Syk in the absence and presence of PLCγ2 (Fig. 5*A*). Consequently, BCAP is phosphorylated by Lyn and forms a complex containing either Syk or Lyn kinases and PLCγ2 irrespective of its activation by kinase.

We also investigated the ability of PLCγ2 to interact with the TIR domain-containing proteins SARM, MAL/TIRAP, and MyD88. Both MyD88 and MAL/TIRAP were able to interact with PLCγ2. In contrast, SARM appears not to associate directly with PLCγ2 (Fig. 5*B*).

## Discussion

Information flow through the TLR pathway is initiated by ligand binding to leucine-rich repeats on the receptor ectodomain followed by receptor dimerization. Subsequently, homotypic associations of cytoplasmic receptor TIR domains and those of various adaptor molecules including MyD88, TRIF, MAL/TIRAP, TRAM, and SARM ensue, setting in motion a cascade of molecular interactions that result in many cellular responses including the production of pro-inflammatory molecules (33). Until recently, the known cytoplasmic TIR-containing adaptors were thought to be involved in direct stimulus-driven TLR signaling only. However, the observation that an adaptor molecule, BCAP, which is involved in BCR signaling, a pathway seemingly unrelated to TLRs, was a negative regulator of inflammation prompted a search for “missing” TIR domains in the human genome and the postulate that BCAP possesses a hidden N-terminal TIR domain (14).

Here we show conclusively that BCAP has an N-terminal TIR domain that structurally bears closest resemblance to the homologous domain in TLR2. The isolated TIR domain of BCAP is monomeric both in the crystal and in solution, a feature shared with most non-plant TIR domains (34), suggesting that vertebrate TIR domain-containing proteins require other domains to promote self-oligomerization: leucine-rich repeats (LRRs) for the TLR, a death domain for MyD88, and the DBB domain and likely the ANK repeats for BCAP. The BCAP_L_ paralogue, BANK1, which regulates CpG-induced IL-6 production (35), also has a TIR domain as the corresponding domains have high sequence homology (40%).

A strong interaction with MAL/TIRAP, TLR4, and TLR2 is also detected in yeast two-hybrid assays. This implies that the dimeric BCAP TIR domain makes multiple interactions with the TLR post-receptor complex. MAL/TIRAP is of particular interest because this adaptor localizes to the plasma membrane by binding to PIP2, an association that is required for signaling (36).

**FIGURE 6.**
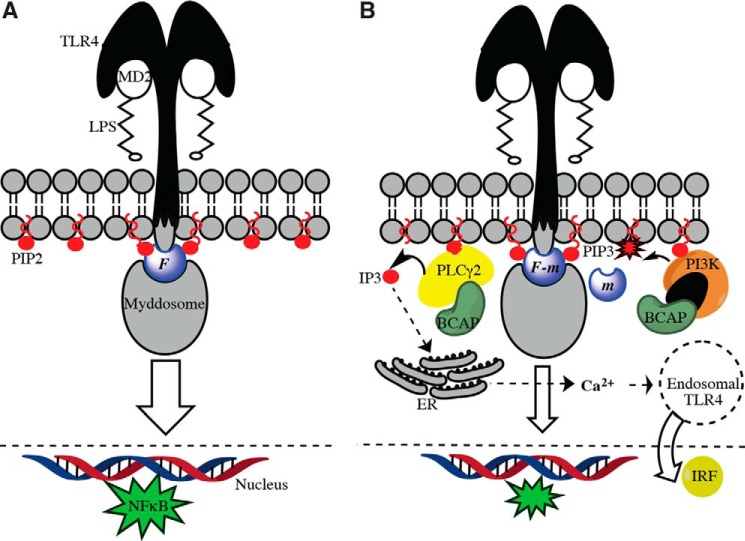
**Negative regulation of TLR4 signaling by BCAP and PLC**γ**2.** *A*, MD2/LPS binding to the TLR4 ectodomain enables the recruitment of a cytosolic signalosome comprising the PIP2-linked adaptor MAL/TIRAP and the Myddosome (MyD88 and interleukin-1 receptor-associated kinase (IRAK) kinases), which interact with the receptor TIR domains. The flux, *F*, through this pathway stimulates the production of NFκB. *B*, PLCγ2-linked BCAP cleaves PIP2 to DAG and IP3, which deprives MAL/TIRAP of its membrane anchor. Additionally, PI3K further depletes PIP2 by converting it to PIP3. MAL/TIRAP is still capable of interacting with PIP3, but it is inactive presumably because it can no longer recruit the Myddosome. The net result is a reduction in flux, (*F* − *m*), and thus negative regulation, of the MD2/LPS-induced stimulation of TLR4. IP3 produced by PLCγ2 cleavage of PIP2 stimulates calcium release from the endoplasmic reticulum (*ER*), which in turn drives the internalization of TLR4 into endosomal compartments, leading to the production of interferon regulatory factors (*IRFs*).

Together our findings provide evidence that BCAP is part of a membrane-bound network that down-regulates inflammatory signaling by the TLRs. Thus, activation of PI3K and PLCγ2 results in the conversion of PIP2 to PIP3 and inositol 1,4,5-triphosphate (IP3), respectively, leading to depletion of MAL/TIRAP from the membrane (Fig. 6*B*) and negative regulation of MAL/TIRAP-dependent TLR signaling.

The association between BCAP and PLCγ2 appears to be more stable than the transient interaction observed between BANK1 and PLCγ2 (37) and may reflect cooperative effects in complex assembly. In contrast, the association between human MAL/TIRAP or MyD88 and PLCγ2 appears to be less stable than that of BCAP with PLCγ2, which likely reflects the central role played by BCAP in modulating all three pathways. Our finding that BCAP interacts directly with PLCγ2 is consistent with published reports linking BCAP to PLCγ2-dependent calcium release (16). Calcium release initiated by the cleavage of PIP2 by PLCγ2 releases diacylglycerol and the potent second messenger IP3, which subsequently interacts with IP3 receptors in the endoplasmic reticulum (38). The resulting calcium flux activates both TLR signaling (17, 39–41) and the trafficking of TLR4 to endosomal compartments (17).

It is also likely that BCAP is the “missing link,” coupling LPS activation of TLR4 to endocytosis and the TRAM/TRIF pathway that induces interferon-β. Zanoni *et al.* (42) have shown that the extrinsic membrane pattern recognition receptor (PRR) CD14 is required for the LPS-induced internalization of the TLR4 post-receptor complex and that this function of CD14 is dependent upon the activation of both Syk and PLCγ2. A further correlation involves the potential role of MAL/TIRAP as a promiscuous lipid-binding protein (43). Although this sorting adaptor had only been implicated in signaling from the cell surface TLR2 and TLR4, Bonham *et al.* (43) present evidence that MAL/TIRAP is required for resident endosomal TLRs such as TLR9, at least in response to natural viral infection rather than synthetic ligands such as phosphorothioate CpG-containing oligonucleotides. Endosomal membranes lack PIP2, but MAL/TIRAP may bind to other phospholipids that are abundant in this compartment such as phosphatidylinositol 3-phosphate (PI(3)P). As noted, BCAP_L_ also negatively regulates endosomal TLRs, perhaps by modulating the levels of these alternative phospholipids in endocytic vesicles. The precise pathways that mediate this crosstalk remain to be elucidated.

There are other scenarios in which PLCγ2 activity is linked to TLR pathway outcomes. PLCγ2 is crucial in the calcium signaling response of TLR2 and TLR4 stimulation by their respective ligands and is also required for the peptidoglycan-dependent production of TNFα and IL-6 (40). Additionally, the calcium flux produced by PLCγ2 is crucial for TRIF-dependent activation of IRF-3 but not the LPS-induced activation of TNFα (17). The activation of PLCγ2 in BCR signaling in murine splenic B-cells is the result of phosphorylation on Tyr-753, Tyr-759, and Tyr-1217 by Btk, but in Ramos cells, phosphorylation of Tyr-1217 is Btk-independent, implying the presence of Btk-dependent and Btk-independent pathways (44). Indeed, its phosphorylation by Syk is implicated in the production of TNFα following stimulation of TLR9 by CpG. BCAP, however, is involved in the production of IL-6 and IL-10 but not TNFα upon TLR stimulation (10). Thus, our finding that the association of BCAP and PLCγ2 is not enhanced by co-expression with Syk is consistent with those observations.

As summarized in Fig. 6, this study partially uncovers a complex membrane-associated network that regulates inflammation by establishing a negative feedback loop between TLR activation and the metabolism of inositol phospholipids. Future studies will elucidate more fully the interactions in this network and the molecular basis of the multitypic interactions mediated by the BCAP TIR domain.

## Experimental Procedures

### 

#### 

##### Protein Expression and Purification for Biophysical Analysis

The N-terminal domain of human BCAP comprising residues 7–142 was cloned into pMCSG7 (DNASU) and expressed as an N-terminal hexahistidine-tagged fusion protein in *E. coli* BL21DE3 cells (Novagen). After nickel-nitrilotriacetic acid purification in 20 mm Tris, 20 mm NaCl, pH 7.5, samples were treated with tobacco etch virus (TEV) protease to remove the hexahistidine tag, and further purification was performed using S200 (GE Healthcare) size exclusion chromatography in the same buffer supplemented with 5 mm DTT. BCAP_L_ (1–805), BCAP_S_, and BCAP_330_ were cloned into the same vector with a Strep-tag at the C terminus. Proteins were purified using a 5-ml StrepTrap column (GE Healthcare), and proteins were eluted using 2.5 mm desthiobiotin (Sigma) dissolved in the same buffer with final purification on S200 size exclusion as described above.

##### Analytical Centrifugation and SEC-MALS

Sedimentation velocity measurements were performed using a Beckman XL-A ultracentrifuge equipped with absorbance and interference optical systems. Sample concentrations ranged from 0.3 to 1 mg/ml. Data were acquired every 300 s at rotor speeds of 30,000–40,000 rpm using an An60Ti rotor (Beckman Coulter). Buffer density and viscosity were estimated using SEDNTERP, and the experimental data were analyzed using SEDFIT (45). SEC-MALS data were obtained using an injection volume of 50 μl and a Superdex 200 Increase 10/300 column (GE Healthcare). Multiangle light scattering and differential refractometry data were acquired using DAWN 8^+^ and Optilab T-rEX instruments, respectively (Wyatt Technology), and data analysis was performed using the ASTRA (v6.1) software package from the manufacturer. Protein concentrations were 4, 2, and 0.8 mg/ml for the TIR, BCAP_330_, and BCAP_L_, respectively.

##### Protein Production for Crystallization

Following nickel-nitrilotriacetic acid purification of the N-terminal domain of BCAP, samples were treated with tobacco etch virus protease to remove the hexahistidine tag and then buffer-exchanged using a Hi-Prep 26/10 desalting column (GE Healthcare) into 50 mm HEPES, 100 mm NaCl, 2 mm DTT, pH 7.5. Following lysine dimethylation (46), samples were purified by size exclusion chromatography into 20 mm TRIS, 20 mm NaCl, 5 mm DTT, pH 7.5. Crystals were obtained using the sitting-drop vapor diffusion method at 20 °C and protein concentrations of 5–7 mg/ml. The well solution contained 100 mm MES, pH 6.5, 2–10% PEG 400, 1.5–2.4 m (NH_4_)_2_SO_4_, and 1 mm 5-amino-2,4,6-triiodoisophthalic acid monohydrate (I3C) (18).

##### Crystallographic Data Collection and Structure Determination

Diffraction data were collected on the IO3 beam-line (Diamond Light Source, Didcot, Oxfordshire, UK). Data reduction and scaling were performed using XDS (47) and Aimless (48). Initial phases using the anomalous signal from I3C were obtained using SHELXD (49), and a partial model was obtained using Buccaneer (50). Further rounds of manual model building and refinement were performed using Coot (51), O (52), and PHENIX (53), respectively.

##### Antibodies

The antibodies used were mouse α-Myc (Santa Cruz Biotechnology), mouse monoclonal α-FLAG M2 (Sigma-Aldrich), rabbit α-FLAG (Sigma-Aldrich), and α-V5-HRP conjugate (Invitrogen).

##### Yeast Two-hybrid Assays

The bait plasmid for yeast two-hybrid assay was generated by inserting the first 434 amino acids of human BCAP (Source Bioscience) in-frame into the pGBKT7 vector (Clontech) to be expressed as a GAL4-DBD fusion protein. The prey plasmids were generated by inserting either the full-length proteins or different protein mutations and truncations in-frame into the pGADT7 vector (Clontech) to be expressed as GAL4-AD fusion proteins. The TIR domain of TLR2 contained residues 636–784, whereas the TIR domain of TLR4 comprised residues 671–820. Protein-protein interactions were assayed by transforming the bait plasmid into the yeast strain AH109 (Clontech) and the prey plasmids into the yeast strain Y187 (Clontech) using standard lithium acetate transformation (54) with the Matchmaker yeast two-hybrid system (Clontech). The Leu+ and Trp+ transformants were selected on SD/−Leu and SD/−Trp plates, respectively. For the interaction analysis, equally dense suspensions of each transformant were prepared in 0.5× yeast extract, peptone, dextrose and adenine broth, mixed, and allowed to mate for 21 h at 30 °C with shaking at 50 rpm. The respective pair of transformants (bait and prey) was then plated onto SD/−Leu/−Trp plates for assessing mating efficiency and on SD/−Leu/−Trp/−His/−Ade+Xα-Gal+ 5 mm 3-amino-1,2,4-triazole (3-AT) plates for assessing the interaction. Negative controls were the empty vectors of pGBKT7 and pGADT7 treated similarly.

##### Pulldown Assays

Human BCAP and splice variant was cloned into either p3XFLAG-CMV-10 or pCMV/Myc vectors. Protein constructs were transfected into HEK 293T cells (ATCC, Manassas, VA) (70–80% confluence) using JetPEI (Polyplus-transfection SA) according to the manufacturer's recommendations. 3 μg of plasmid DNA was transfected per well in a 6-well plate. When required, pcDNA3.1 was included to ensure that the total DNA transfected was 3 μg. The transfection mixture was incubated for 24 h, after which cells were washed once in 1× PBS and lysed using 300 μl of a solution containing 20 mm Tris, 150 mm NaCl, 1 mm EDTA, 0.5% Nonidet P-40, pH 8.0, which was supplemented with 1% Triton X-100, 0.5% *n*-octylglucoside, 1 mm PMSF, and 1× protease inhibitor cocktail (Calbiochem). After incubation on ice for 10 min, the mixture was transferred to an Eppendorf tube, and lysis was continued for an additional 30 min at 4 °C with agitation, after which the mixture was centrifuged at 16,000 × *g* for 10 min at 4 °C, and the supernatant was collected for analysis by Western blotting or for immunoprecipitation with anti-FLAG M2 beads (EZview Red anti-FLAG M2 affinity gel, Sigma). 250 μl of the extracted protein sample was incubated with anti-FLAG beads for 2 h at 4 °C with constant rotation, after which non-specifically bound proteins were removed by repeated centrifugation at 1000 × *g* and resuspension of the beads with 500 μl of lysis buffer. After the final wash, excess solution was aspirated, and 30 μl of 1× SDS loading buffer was added to the beads and heated at 100 °C for 10 min. Finally, the protein samples from whole cell lysate and immunoprecipitation were analyzed by Western blotting using the indicated antibodies.

## Author Contributions

S. H. produced proteins, conducted Y2H and pulldown experiments and assisted with the AUC and crystallographic analysis and writing. E. S. and B. V. supplied the data for supplemental Fig. 3. M. C. M. wrote most of the paper and performed AUC analysis and X-ray structure determination and SEC-MALS. N. J. G. initially conceived the project and helped with writing the paper.

## Supplementary Material

Supplemental Data

## Footnotes

4 The abbreviations used are:

BCAP: B-cell adaptor for phosphoinositide 3-kinase
TLR: Toll-like receptor
TIR: Toll/interleukin-1 receptor
Btk: Bruton's tyrosine kinase
IP3: inositol 1,4,5-triphosphate
PIP2: phosphatidylinositol 4,5-bisphosphate
PIP3: phosphatidylinositol 1,4,5-trisphosphate
BCR: B-cell receptor
PLCγ: phospholipase Cγ
I3C: 5-amino-2,4,6-triiodoisophthalic acid monohydrate
AUC: analytical ultracentrifugation
SEC-MALS: size exclusion chromatography with multiangle light-scattering analysis
FL: full-length.
