# Supplementary material for: Structure of the Toll/Interleukin-1 Receptor (TIR) Domain of the B-cell Adaptor That Links Phosphoinositide Metabolism with the Negative Regulation of the Toll-like Receptor (TLR) Signalosome
Source: J Biol Chem. 2016 Dec 1;292(2):652–60. doi: 10.1074/jbc.M116.761528 (PMC5241739; doi:10.1074/jbc.M116.761528)
Supplement: Supplemental Data [file supp_292_2_652__index.html]

Structure of the TIR domain of BCAP which links phosphoinositide metabolism with the negative regulation of the TLR signalosome — Structure of the Toll/Interleukin-1 Receptor (TIR) Domain of the B-cell Adaptor That Links Phosphoinositide Metabolism with the Negative Regulation of the Toll-like Receptor (TLR) Signalosome — TIR Domain of the BCAP Adaptor — Supplemental Data 

# Structure of the Toll/Interleukin-1 Receptor (TIR) Domain of the B-cell Adaptor That Links Phosphoinositide Metabolism with the Negative Regulation of the Toll-like Receptor (TLR) Signalosome

## Supplemental Data

- supp. data (.pdf, 1.8 MB) - supplementary data with new figure
