# Supplementary material for: Structure of the Toll/Interleukin-1 Receptor (TIR) Domain of the B-cell Adaptor That Links Phosphoinositide Metabolism with the Negative Regulation of the Toll-like Receptor (TLR) Signalosome
Source: J Biol Chem. 2016 Dec 1;292(2):652–60. doi: 10.1074/jbc.M116.761528 (PMC5241739; doi:10.1074/jbc.M116.761528)
Supplement: Supplemental Data [file 10.1074_M116.761528_jbc.M116.761528-1.pdf]

SUPPLEMENTARY MATERIALS

Supplementary Table S1: Crystallographic Statistics for the N-terminal TIR domain of human BCAP

	**BCAP-TIR (I3C)**
**Data Collection**	
Space group	P62 2 2
Cell dimensions	
$a = b, c$ (Å)	135.04, 42.91
Resolution (Å)	44.2–2.5
Total reflections	99543 (9785)
Unique reflections	15066 (1489)
$I/\sigma I$	24.8(7.2)
Completeness (%)	99(98)
Redundancy	6.6(6.6)
Wilson B-factor	53.84
R-merge	0.047 (0.24)
R-meas	0.051 (0.26)
CC1/2	0.99 (0.98)
CC*	1.0 (1.0)
Phaser-EP FOM	0.35
**Refinement**	
No. reflections	15066 (1489)
R_{work}/R_{free}	0.19/0.24
No. atoms	
Protein	1088
Ligands	32
Avg. B-factor	59.7
R.m.s. deviations	
Bond lengths (Å)	0.012
Bond angles (Degrees)	1.60
**Validation**	
Ramachandran favoured/allowed (%)	96/100
Rotamer Outliers (%)	7.8
Clashscore	7.7

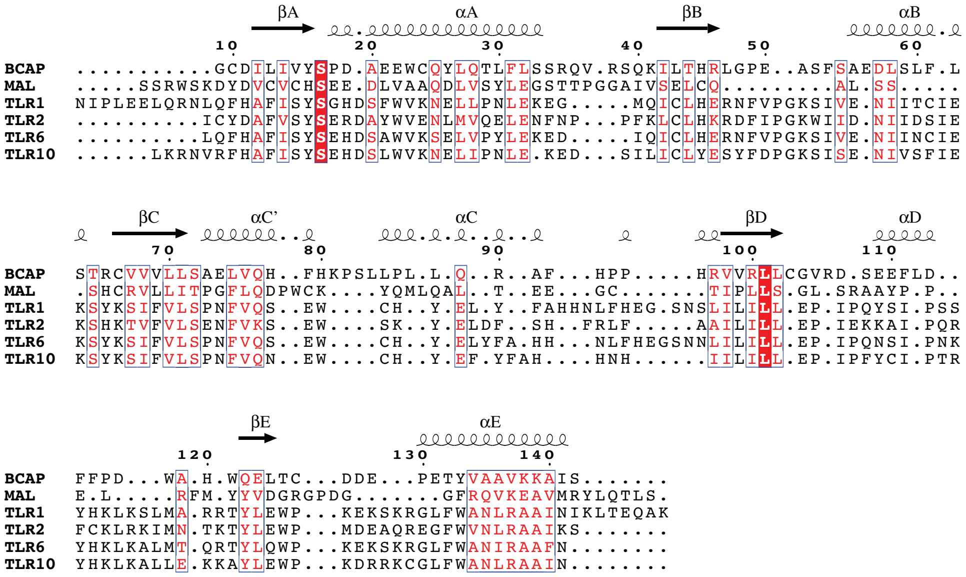

Supplementary Fig. S1: Structural alignment of the BCAP-TIR and the TIR-domains of MAL (2y92), TLR1 (1fyv), TLR2 (1o77), TLR6 (4om7) and TLR10 (2j67). The secondary structural elements shown are those of the BCAP-TIR domain (5for).

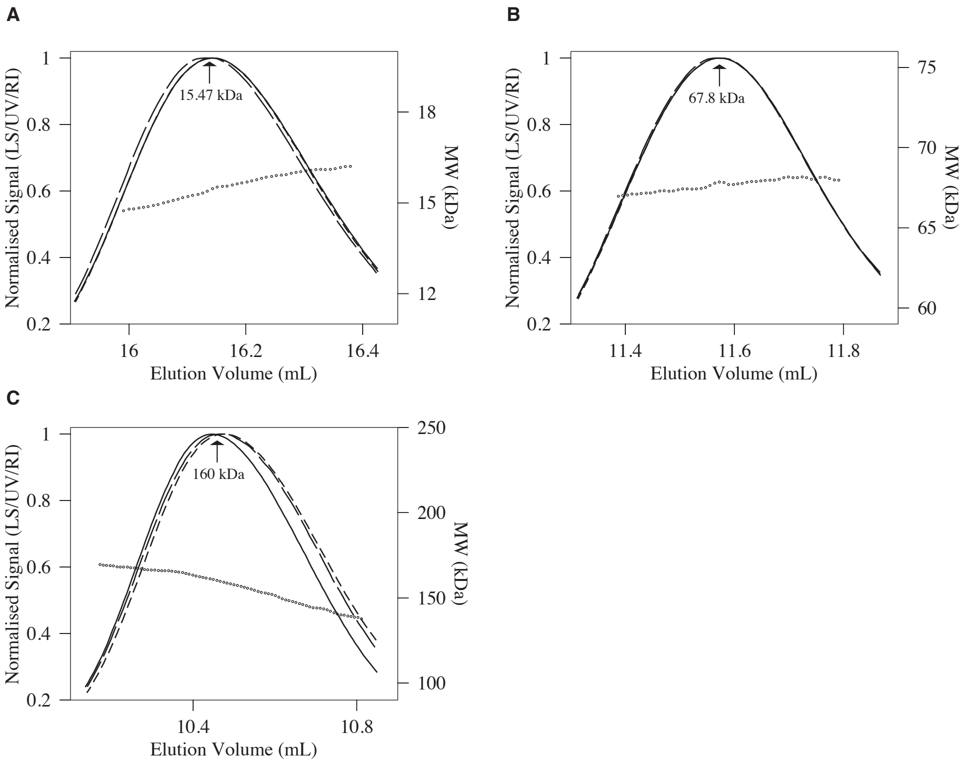

Supplementary Fig. S2: Size exclusion chromatography in combination with multi-angle light scattering (SEC-MALS) and differential refractometry data for the N-terminal TIR domain of BCAP (A), $BCAP_{330}$ (B) and $BCAP_{L}$ (C). Plots show the normalised UV (280 nm) light scattering, refractive index and weight-averaged molecular mass (MW) variation across the peak in addition to the MW at the peak. The molecular weights obtained by SEC-MALS are in complete agreement with those obtained by sedimentation velocity.)

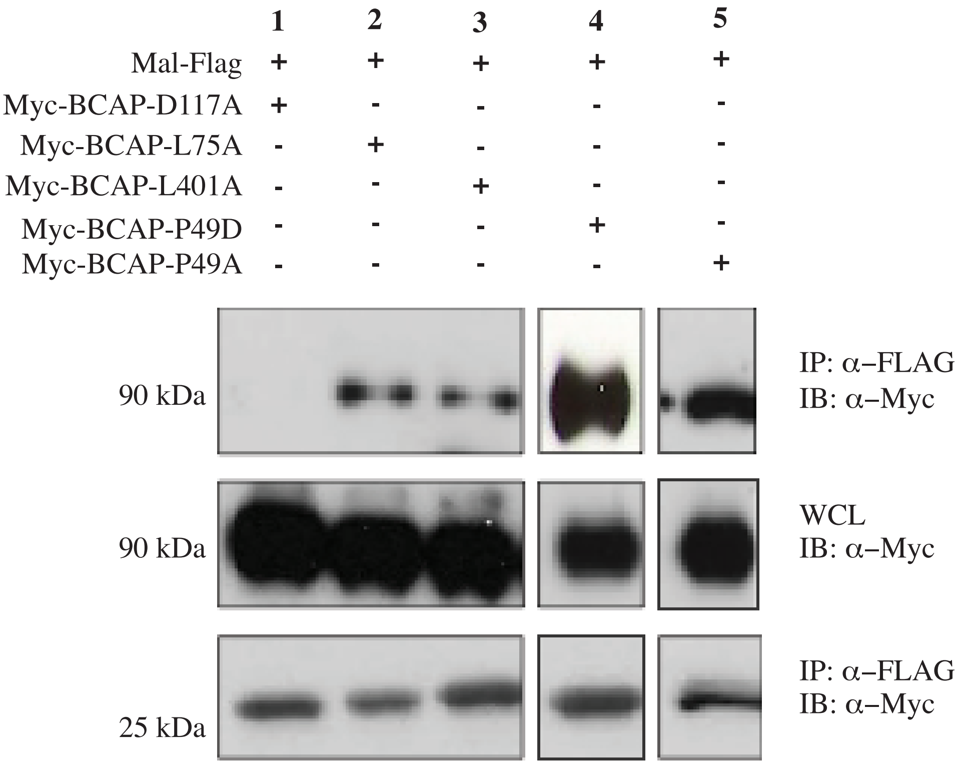

Supplementary Fig. S3: The highly conserved proline residue (P49) in the βB-loop of BCAP is not required for Mal/TIRAP interaction. HEK293T cells were transfected with FLAG-Mal, Myc-Mal and Myc-BCAP as described. Both βB loop proline mutations (P49A/P49D) allowed the BCAP-Mal/TIRAP interaction (lanes 4–5) to occur. Mutations in the αC′ helix (L75A) and outside the TIR domain (L401A) both exhibit reduced interaction with Mal/TIRAP (lanes 2–3) while the D117A (lane 1) mutation which lies in the region connecting the αD helix and βD strand completely abolishes the BCAP and Mal/TIRAP interaction.

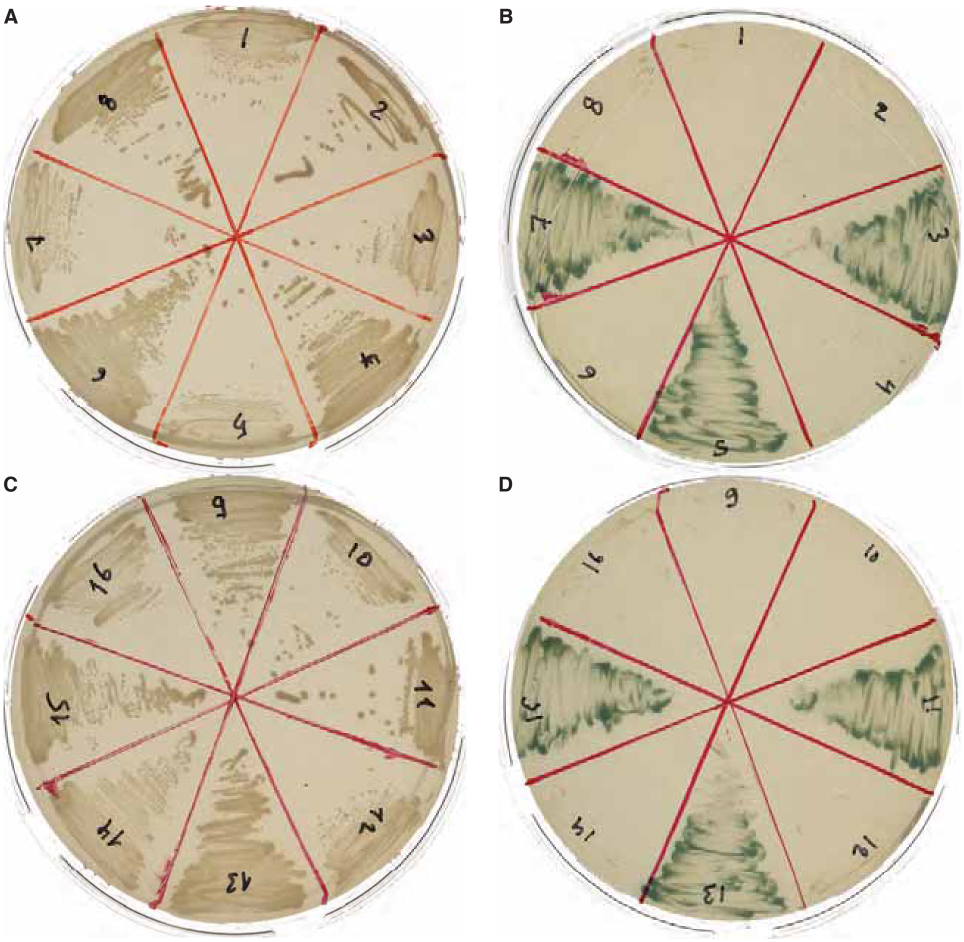

Supplementary Fig. S4: Interaction of BCAP-TDA and various TIR domain containing proteins determined by Yeast two-hybrid. Panels (A) and (C) were used to access transformation and mating efficiency on SD/-Trp/-Leu media. BCAP-TDA interacts with wild-type MAL/TIRAP and its mutants D96N and S180L (3, 5 and 7)(B) and with the TIR domains of TLR2 and TLR4 (D) (11 and 13) respectively on SD/-Trp/-Leu/-His/-Ade/+X-α-Gal+5mM 3-AT. The full matrix of the prey and bait plasmids used are shown below.

	pGADT7	pGADT7[TIRAP]	pGADT7[D96N]	pGADT7[S180L]
pGBKT7[BCAP-TDA]	1	3	5	7
pGBKT7	2	4	6	8
	pGADT7	pGADT7[TLR2]	pGADT7[TLR4]	pGADT7[TIRAP]
pGBKT7[BCAP-TDA]	9	11	13	15
pGBKT7	10	12	14	16

Supplementary Table S2: Plasmids and primers used for protein expression in bacteria, yeast and human cells. The restriction sites used, when relevant are shown.

Protein	Oligonucleotide Sequence (5′ → 3′)	Vector
BCAP-TIR(F)	TACTTCCAATCCAATGCCCCCAGAGGATGCGACATCCTCATC	pMCSG7
BCAP-TIR(R)	TTATCCACTTCCAATGTTAGGAAATGGCTTTTTCACAGCTGCCAC	
$BCAP_S$(F)	TACTTCCAATCCAATGCCATGGTGGTGCAGCCGGACCG	pMCSG7
$BCAP_{L/S}$(R)	TTATCCACTTCCAATGTTACTTCTCGAACTGGGGGTGACTC	pMCSG7
	CAGCGTCCTCTGGGTGG	
$BCAP_{L/330}$(F)	TACTTCCAATCCAATGCCATGGCAGCCTCAGGG	pMCSG7
$BCAP_{330}$(R)	TTATCCACTTCCAATGTTACTTTTCAAACTGCGGATGCGACCAG	
	GCACTGGCATTTGTCATCATATCTTCTTCTTCCAGCTGGTTG	
FLAG-$BCAP_L$(F)	ATCAAGCTTATGGCAGCCTCAGGGGTGCC (HindIII)	pCMV10
FLAG-$BCAP_L$(R)	ATCGGATCCTCAGCGTCCTCTGGGTGGAACAGG (BamHI)	
Myc-$BCAP_L$ (F)	Same as FLAG-$BCAP_L$(F) (HindIII)	pCMV-Myc
Myc-$BCAP_L$ (R)	ATCGGTACCGCGTCCTCTGGGTGGAACAGG (KpnI)	
Myc-BCAP-SV (F)	ATCAAGCTTATGGTGGTGCAGCCGGACCG (HindIII)	pCMV-Myc
Myc-BCAP-SV (R)	ATCGGTACCGCGTCCTCTGGGTGGAACAGG (KpnI)	
BCAP-TDA (F)	ATCCATATGATGGCAGCCTCAGGGGTGCC (NdeI)	pGBKT7 AD
BCAP-TDA (R)	ATCGGATCCTCACGAGCATTTCATAAGCAGGTCTGTGG (BamHI)	
MAL (F)	ATCCATATGATGGCATCATCGACCTCCCTCCC (NdeI)	pGBKT7 AD
MAL (R)	ATCGGATCCTCAAAGTAGATCAGATACTGTAGCTGAATCCCG (BamHI)	
TLR2-TIR (F)	ATCCATATGAGCAGGAACATCTGCTATGATGCATTTGTTTC (NdeI)	pGBKT7 AD
TLR2-TIR (R)	ATCGGATCCTCAGGACTTTATCGCAGCTCTCAGATTTACC (BamHI)	
TLR4-TIR (F)	ATCCATATGGAAAACATCTATGATGCCTTTGTTATCTACTCAAG (NdeI)	pGBKT7 AD
TLR4-TIR (R)	TCGGATCCTCATGATTTACCATCCAGCAGGGCTTTTCTG (BamHI)	
